# Seed-Derived Ethylene Facilitates Colonization but Not Aflatoxin Production by *Aspergillus flavus* in Maize

**DOI:** 10.3389/fpls.2017.00415

**Published:** 2017-03-28

**Authors:** Shi Wang, Yong-Soon Park, Yang Yang, Eli J. Borrego, Tom Isakeit, Xiquan Gao, Michael V. Kolomiets

**Affiliations:** ^1^State Key Laboratory of Crop Genetics and Germplasm Enhancement, College of Agriculture, Nanjing Agricultural University,Nanjing, China; ^2^Jiangsu Collaborative Innovation Center for Modern Crop Production, Nanjing Agricultural University,Nanjing, China; ^3^Department of Plant Pathology and Microbiology, Texas A&M University, College Station,TX, USA; ^4^Division of Biotechnology, Chonbuk National University,Iksan, South Korea

**Keywords:** *Aspergillus flavus*, ethylene, maize, colonization, aflatoxin

## Abstract

Ethylene (ET) emitted by plant tissues has been broadly reported to play important roles in plant development, response to environmental stresses and defense against certain pathogens. Recent evidence obtained from using *in vitro* fungal cultures exposed to ET suggested that exogenous ET may regulate the production of aflatoxin by *Aspergilli*. However, the function of endogenous, seed-derived ET has not been explored. In this study, we found that the maize lipoxygenase *lox3* mutant, previously reported to be susceptible to *Aspergillus* spp., emitted greater levels of ET upon *A. flavus* infection, suggesting the potential involvement of endogenous ET in the susceptibility of maize to *A. flavus*. Supporting this idea, both colonization and conidiation of *A. flavus* were reduced in wild-type (WT) kernels treated with AgNO_3_, an ET synthesis inhibitor. There was no ET emission from non-viable kernels colonized by *A. flavus*, suggesting that living seed but not the fungus itself was the primary source of ET released upon infection with *A. flavus*. The kernels of *acs2* and *acs6*, two ET biosynthetic mutants carrying *Mutator* transposons in the ACC synthase genes, *ACS2* and *ACS6*, respectively, displayed enhanced seed colonization and conidiation, but not the levels of aflatoxin, upon infection with *A. flavus*. Surprisingly, both *acs2* and *acs6* mutant kernels emitted greater levels of ET in response to infection by *A. flavus* as compared with WT seed. The increased ET in single mutants was found to be due to overexpression of functional ACS genes in response to *A. flavus* infection. Collectively, these findings suggested that ET emitted by infected seed facilitates colonization by *A. flavus* but not aflatoxin production.

## Introduction

*Aspergillus* is one of the most common mycotoxin producing fungi that contaminate a large number of crops, both pre- and post-harvest. Some of the major economic losses are caused by aflatoxin (produced by *A. flavus*) contamination of maize and other oil rich seed crops worldwide. Aflatoxin is one of the most potent natural carcinogens hazardous to health of humans and animals when consumed as food or feed, respectively. Unfortunately, conventional agronomic approaches have limited effectiveness for reducing mycotoxin contamination. The ideal solution would be to decrease contamination by improving genetic resistance of these crop plants. Sources of resistance that limit the ability of *Aspergillus* spp. to grow, reproduce and synthesize mycotoxins have been identified. However, despite significant efforts by public and private breeding programs, adequate levels of resistance have not been achieved primarily due to lacking major single resistance genes against those fungi Munkvold (2003). One strategy to accelerate resistance breeding efforts is to combine the approaches of genome-wide association analysis (GWAS) and traditional linkage mapping analysis to identify the *A. flavus* resistance quantitative trait loci (QTL) or genes, and closely linked markers. Recently, a linkage mapping was performed using 228 recombinant inbred lines (RILs) of maize, and a highly significant QTL that affected aflatoxin accumulation, qAA8, was mapped to chromosome 8, which can explain 6.7 to 26.8% of the phenotypic variation (Zhang et al., 2016). Several other large QTLs have also reported to be located on chromosomes one, three, four, five, and nine (Xiang et al., 2010; Warburton et al., 2011; Willcox et al., 2013). Another promising approach to aid conventional breeding efforts is to identify genes and signaling pathways regulated by these genes that underlie host resistance (or susceptibility) mechanisms. This information can be used to develop and breed beneficial alleles of these genes or genetic engineering approaches to control infection and mycotoxin production.

One of the plant-derived molecular signals that have been implicated in the regulation of aflatoxin biosynthesis is a gaseous plant hormone ethylene (ET). In higher plants, ET is initially synthesized from methionine (Met) via S-adenosyl-L-methionine (S-AdoMet) by SAM synthetase. S-AdoMet is subsequently converted to 1-aminocyclopropane-1-carboxylate (ACC) by ACC synthase (ACS, S-adenosyl-L-methionine methylthioadenosine-lyase) and finally to ET by ACC oxidase (ACO) (Yang, 1985; Kende, 1993). As a by-product of this reaction, ACS also produces 5′-methylthioadenosine (5′-MTA), which is recycled in the Yang Cycle to synthesize methionine (Met), to maintain the constant level of the cellular Met for continuous protein and ET biosynthesis (Yang, 1985; Bleecker and Kende, 2000).

Besides higher plants, ET is also synthesized by micro-organisms, including phytopathogenic fungi and bacteria (Fukuda et al., 1993), through either 2-keto-4-methylbutyric acid (KMBA) as reported for *Escherichia coli* (Ince and Knowles, 1986) and *Cryptococcus albidus* (Fukuda et al., 1989b), or via 2-oxoglutarate by *Penicillium digitatum* (Fukuda et al., 1989a) and *Pseudomonas syringae* (Nagahama et al., 1991). The fungus *Penicillium citrinum* can also synthesize ACC from SAM (Jia et al., 1999), while *Botrytis cinerea* appears to utilize the KMBA pathway to produce ET (Cristescu et al., 2002; Chague et al., 2006). However, the physiological significance of ET production by these microorganisms has not been well studied, but is hypothesized to facilitate pathogen virulence.

In plants, ET has been broadly reported to play important roles in regulating diverse physiological and defense processes, including seed germination, organ senescence, abscission and fruit ripening (Kende, 1993; Johnson and Ecker, 1998), and responses to abiotic and biotic stresses, such as wounding, chilling, drought, flooding, hypoxia, ozone damage and pathogen attack (Paul et al., 2003; Lin et al., 2009; Musungu et al., 2016). ET modulates various defense responses against pathogens either individually or in combination with other phytohormones (Guo and Ecker, 2004; Broekaert et al., 2006; Adie et al., 2007; Kendrick and Chang, 2008). For instance, the resistance of plants to the necrotrophic fungal pathogen *B. cinerea* was moderately enhanced by exogenous treatment of plants with ET (Diaz et al., 2002). Also, ET biosynthesis is activated when plants are challenged by pathogens; on the other hand, increased ET production is associated with enhanced defense-related gene expression (Penninckx et al., 1998; Cohn and Martin, 2005). ET acts synergistically with jasmonates (JAs) in defense responses (Penninckx et al., 1998; Ellis and Turner, 2001; Catinot et al., 2015), often accompanied by induction of clusters of genes regulated by ET or jasmonic acid (JA) (Glazebrook et al., 2003; Broekaert et al., 2006).

In addition to acting as a signaling molecule in plant defense against pathogens, ET has also been implicated in the regulation of mycotoxin production by *Aspergillus* spp. When ET was exogenously applied to peanuts inoculated with *A. parasiticus*, aflatoxin accumulation was significantly reduced (Roze et al., 2004; Gunterus et al., 2007; Huang et al., 2009). An ET generator, 2-chloroethyl phosphoric acid (CEPA, ethephon), also suppressed aflatoxin biosynthesis in *A. flavus*, and this suppression was probably due to the reduction of reactive oxygen species (ROS) (Huang et al., 2009). These data suggest a role for exogenous ET in modulating secondary metabolism of *Aspergillus* spp. However, it remains to be explored whether and how the endogenous ET derived from host plants impacts the fungal colonization and mycotoxin production. In this study, the role of seed-derived ET in maize interactions with *A. flavus* was elucidated by kernel bioassay using maize mutants disrupted by transposon insertions in the *ACS2* and *ACS6* genes.

## Materials and Methods

### Plant Materials and Fungal Strains

Maize *acs* mutants (*acs2* and *acs6*) and lipoxygenase *lox3-4* mutants were generated by *Mutator-*transposable element-insertional mutagenesis as described previously (Young et al., 2004; Gao et al., 2007). These *acs* mutants were backcrossed seven times into B73 background resulting in generation of mutants that are near-isogenic to the recurrent parent line B73. The *lox3-4* mutants are at the BC_5_F_3_ genetic stage. In all experiments, *lox3-4* mutants were compared to near-isogenic WTs obtained by self-pollinating WT siblings identified in the BC_5_F_2_ segregating population.

To measure the expression levels of *ACS2*, *6* and *7* in different organs, the samples were collected from different organs at different stages. For geminating stage, the embryos and roots were excised from the 2-day-old or 4-day-old germinated seeds, respectively. For seedling stage, roots, stems and leaves were harvested from V1 and V3 stages grown in a light shelf, respectively. For adult stage, roots, stems, leaves, pollens, tassels and ears were harvested from the matured plants. The seeds after completely matured were also harvested for gene expression study at matured stage.

*Aspergillus flavus* NRRL 3357 was cultured at room temperature on potato dextrose agar (PDA: Difco) as described previously (Gao et al., 2009).

### Fungal Inoculation and Spore Counting on Kernels

Maize kernels in similar size were selected and weighed to ensure equivalent average seed weights across all samples. Seeds were then surface-sterilized with 100% Clorox bleach (containing 6% sodium hypochlorite) for 10–15 min and rinsed with sterilized, distilled H_2_O at least five times. The embryos of kernels were cut longitudinally using a razor blade to a depth of about 0.5 cm to provide an infection court for fungal inoculation. Seeds were then blotted dry with paper towel and placed in a 20-ml glass-scintillation vial (Wheaton Science, Millville, NJ, USA) and inoculated with 200 μl of conidia suspension (1 × 10^6^/ml) of *A. flavus* NRRL 3357. Conidia were harvested with 0.001% Tween 20 from fungal strains grown in PDA plate. Control seeds (mock) received equal amount of 0.001% Tween 20. Four or six inoculated or mock-treated kernels were used per replicate with at least four replicates per experiment. The inoculated kernels were kept in a plastic transparent container with a wet filter paper to provide humidity and incubated with 12 h light/day at 26–29°C. Sterile, distilled H_2_O was added to containers as needed to maintain high humidity. Kernels were harvested at designated intervals after inoculation, either to enumerate conidia or to quantify mycotoxins.

To measure levels of conidia production, infected kernels were placed in a 20 mL glass vial with 2 mL of 0.001% Tween 20, and vortexed for 20 s to dislodge spores. The spore suspension was decanted and spores were enumerated using a hemacytometer.

### Semi Quantitative RT-PCR and qRT-PCR for Gene Expression

B73 genetic background kernels were sterilized and inoculated with mock control, *A. flavus*. All seeds were applied with either control or fungi as described above. Control or fungi-challenged seeds were harvested at 0, 12, 24, 48, and 96 h after inoculation. Total RNA was extracted by using TRI reagent (Molecular research Center Inc., Cincinnati, OH, USA) following the manufacturer’s protocol. After extracting total RNA, these RNA samples were treated with RNase-free rDNase at 37°C for 30 min using a DNA-free kit (Ambion Inc., Austin, TX, USA). First strand cDNA synthesis (5 μg of RNA as a template for each sample) was carried out by using a First-Strand Synthesis Kit (GE Healthcare Bio-Sciences Corp., Piscataway, NJ, USA) following the manufacturer’s protocol. The synthesized cDNA was diluted and equalized for all samples. The cDNA as a template was amplified with two gene-specific primers for each gene, and for the house-keeping gene *ZmGAPc*. The cDNA was denatured at 94°C for 5 min and amplified by following 27–32 cycles (each cycle: 45 s at 94°C, 1 min at 56°C, and 2 min at 72°C). Amplified PCR products were loaded and separated on 1.2–1.5% agarose gels.

Quantitative reverse transcription-polymerase chain reaction (qPCR) assay was performed using qRT-PCR kit purchased from Takara (Takara, Japan). Reactions were optimized for RNA and primer concentrations with each 10 μl reaction consisting of 40 ng of DNase-free RNA and 200 nM primers. qPCR analysis was performed in the ABI Prism 7000 system (Applied Biosystems, USA). The program used was as follows: 94°C for 1 min; followed by 40 cycles of 94°C for 5 s, 65°C for 15 s and 72°C for 30 s. Primers used in this study are described in Supplementary Table 1, and Tublin gene was used as internal control. The quantification of gene expression was repeated at least three times.

### Quantification of Aflatoxin and Ergosterol

Infected or mock-treated kernels from each treatment were ground using a Waring blender (Waring laboratory, Torrington, CT, USA), and aflatoxin was subsequently quantified with a fluorometer using the VICAM AflaTest^®^ USDA-FGIS procedure (VICAM, Watertown, MA, USA) with six infected kernels per replicate were frozen in liquid nitrogen until assayed. Ergosterol was extracted from infected kernels overnight with 5 ml chloroform:methanol (2:1 v/v) at room temperature as described by previous study (Woloshuk et al., 1989) with some modifications. Ergosterol was analyzed on a Shimatzu LC-20AT HPLC system (Shimatzu Scientific Instruments, Inc., Kyoto, Japan) equipped with a 4.6 U ODS column (250 mm × 4.6 mm) and a UV detector (282 nm). Quantities were calculated by comparing HPLC peak areas with ergosterol standards (Sigma). The experiment was repeated at least four times, with consistent results.

### Measurement of Ethylene Produced by Kernels, Leaves and Pathogens

ET produced by infected and by control kernels was quantified as described by Gao et al. (2008) with some modifications. Briefly, the vials containing infected kernels were kept at 12 h light/day at 26–29°C and ET was measured at 1, 2, 4, and 7 days post-inoculation (dpi). Vials were sealed with screw caps with septa. The headspace gas (1 ml) was withdrawn from vials by a syringe and analyzed using gas chromatography.

To measure the ET levels produced in the leaves of B73 and *acs6* mutant, the plants were grown at 25 to 28°C in commercial soil (Metro-Mix 366; Scotts-Sierra Horticultural Products, Marysville, OH, USA) under 14 h of daylight with 120 μM m^−2^ s^−1^ (Quantum Meter; Apogee Instruments, Logan, UT, USA). The seedlings were grown in long conical tubes (20.5 by 4 cm) for 2 to 3 weeks until they had three fully expanded leaves (V3 developmental stage). The leaves of B73 and *acs6* were excised from the plants and immediately transferred into a 20 ml of glass scintillation vial (Wheaton Science). The vials were then tightly sealed with a plastic lid for 1 to 2 h prior to analysis to allow enough ethylene was accumulated. ET was measured as described by Gao et al. (2008).

### Treatment with Ethylene Inhibitor and Precursor ACC

The pretreatment of kernels with ET inhibitor AgNO_3_ was performed by adding 1 ml of AgNO_3_ (Sigma-Aldrich, St. Louis, MO, USA) at 20 mM in 0.001% Tween-20, to the freshly wounded kernels as described above (four kernels per vial per replicate) and mixed thoroughly to ensure the complete soaking of kernels with the chemical. At least four replicates were used per treatment/genotype combination. Control kernels received the same volume of 0.001% Tween-20. The kernels were incubated for 30 min and blotted dry to remove excessive solution and were subsequently inoculated with *A. flavus* suspensions as described.

For the treatment with ET precursor, ACC, the kernels were pretreated with ACC (20 nM) in 0.001% Tween-20 for 30 min, followed by inoculation with 1 × 10^6^ spores, then cultured and analyzed as described above. Pretreatment with same amount of 0.001% Tween-20 was used as control.

## Results

### ET Production Is Enhanced in *A. flavus*-Inoculated *lox3-4* Mutant Kernels

We have previously reported that mutation of a maize 9-lipoxygenase gene, *ZmLOX3*, resulted in the increased susceptibility of kernels to the infection with *A. flavus* and *A. nidulans* (Gao et al., 2009). In an earlier study, we found that the *lox3-4* mutants produced greater levels of ethylene in the roots compared to the WT (Gao et al., 2008), which prompted us to investigate whether ET production was also altered in the *lox3-4* mutant kernels in response to *A. flavus* infection.

**FIGURE 1:**
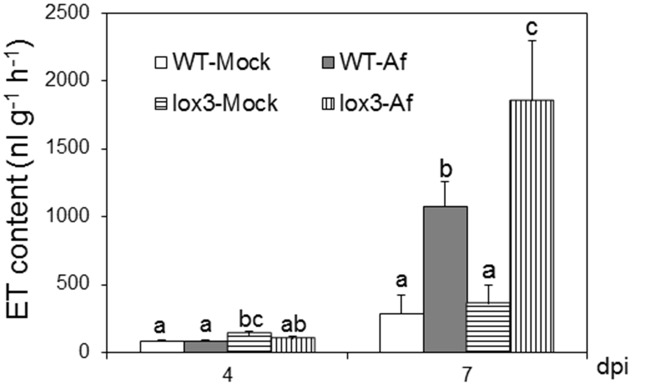
**Ethylene production on *lox3-4* mutants upon inoculation with *A. flavus* NRRL 3357.** The kernels were inoculated with 1 × 10^6^ spores and cultured in a 20 ml of glass scintillation vial (Wheaton Science) at 29°C under 12L/12D for 4 and 7 days. ET was measured by withdrawing 1 mL volume of headspace gas with a syringe and injecting into a digital gas chromatograph (Photovac 10 plus, PerkinElmer, Inc., Norwalk, CT, USA) The values are the mean ± SD of three replicates (four vials each treatment each genotype), with six kernels in each vial. Different letters above bars denote significant differences (*P*< 0.05, ANOVA) between genotypes and treatments within same time point.

**FIGURE 2:**
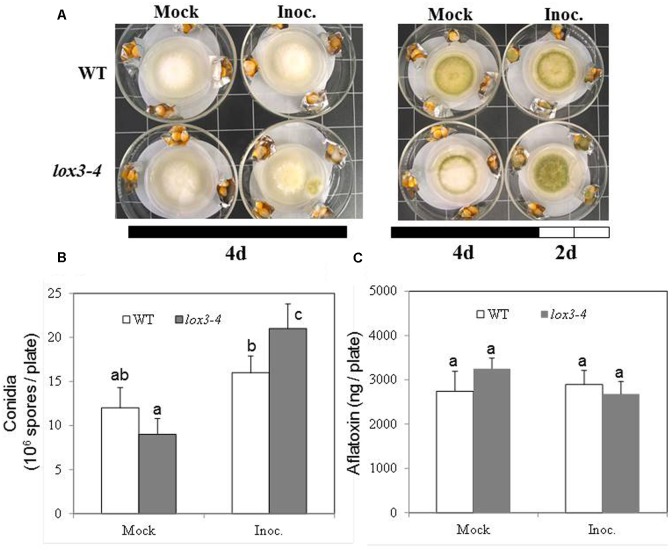
**Kernel plate assay for testing the effect of potent gas produced by *lox3-4* kernels inoculated with *A. flavus* on the fungal growth, sporulation, and aflatoxin production by *A. flavus* grown on a plate.** WT and *lox3-4* kernels were inoculated with 1 × 10^6^ spores and placed in a large petri dish, containing a smaller petri dish where the *A. flavus* was growing on the PDA media **(A)**. **(A)** The infected kernels were co-incubated with the fungal plate for either 4 days under continuous darkness or 4 days continuous darkness then moving to the continuous light for an additional 2 days. **(B)** The conidia, and **(C)** aflatoxin produced from the fungi grown on the PDA plates inside the small petri dishes was determined, respectively, at 6 days post culture (4 days dark+2 days light). The values are the mean ± SD of three replicates, and different letters above bars denote significant differences (*P* < 0.05, ANOVA) between genotypes and treatments.

As shown in **Figure 1**, infection with *A. flavus* substantially increased ET emission in both near-isogenic WT and *lox3-4* mutant, with the latter significantly more than the former at 7 dpi. Because *lox3-4* mutant is substantially more susceptible to *A. flavus* colonization (Gao et al., 2009), we reasoned that one potential mechanism underlying increased susceptibility of the mutant is increased ET. To test whether volatiles including ET emitted by infected kernels impact fungal growth, sporogenesis and toxin production, we performed an *in vitro* plate assay by co-incubating *A. flavus* grown on PDA media in close proximity with maize kernels infected with *A. flavus* simultaneously (**Figure 2A**). The plates were cultured under constant dark condition for 4 days to allow for vegetative growth but prevent sporulation, then transferred to light/dark cycle (8 h dark/16 h light conditions) for another 2 days, This will allow us to investigate whether synchronizing the switch from vegetative growth to asexual reproduction is regulated by exposure to volatiles produced from infected kernels. Exposure to light/dark regimes is required for conidia production as evidenced by rapid green pigmentation of fungal hyphal mass in *Aspergilli* spp. (Rodriguez-Romero et al., 2010; Ruger-Herreros et al., 2011). Conidia and aflatoxin produced by the fungal cultures in response to volatiles emitted by the infected WT or *lox3-4* mutant kernels were measured. As shown in **Figures 2B,C**, after expose to light for 2 days, in contrast to the colonies co-incubated with infected WT kernels, fungal colonies exposed to volatiles emitted by infected *lox3-4* kernels produced greater levels of conidia as evidenced by green pigmentation of fungal colonies and conidia enumeration. However, aflatoxin content produced by *A. flavus* was not different among the plates supplemented with *lox3-4* or WT.

### Ethylene Biosynthetic Genes Are Differentially Induced in Seed in Response to Infection with *A. flavus*

**FIGURE 3:**
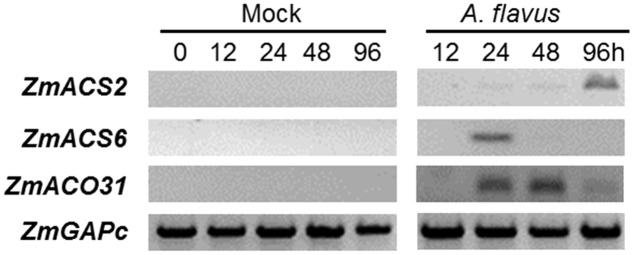
**Genes encoding ethylene biosynthesis enzymes, ACC synthase (*ZmACS*) and ACC oxidase (*ZmACO*) are induced in maize seed in response to *A. flavus* infection.** The WT kernels (B73 inbred line) were inoculated with *A. flavus* or mock-inoculated, incubated and harvested at 0, 12, 24, 48, and 96 h post-inoculation. The expression of two *ACS* genes, *ZmACS2* and *ZmACS6*, and *ZmACO31* were tested. Expression of a house-keeping gene *ZmGAPc* was used as an internal standard in this experiment.

To investigate the role of ET and ET-biosynthesis genes in the regulation of seed colonization of maize by *A. flavus*, we inoculated the WT kernels (B73 inbred line) with fungal spore suspension of *A. flavus* at 1 × 10^6^ spores/ml or H_2_O as a mock control, and the transcript levels of three ET biosynthesis genes, *ZmACS2*, *ZmACS6*, and *ZmACO31* (ACC oxidase 31), were quantified in either mock-treated or *A. flavus* inoculated kernels at different time points post treatments. While the expression of *ZmACO31* was found to be induced in the kernels infected with *A. flavus* at 24 and 48 hpi, the transcriptional level of *ZmACS2* and *ZmACS6* was induced at 96 and 24 hpi, respectively (**Figure 3**). These results suggest that seed respond to pathogen infection by increased synthesis of ET.

To test whether ET biosynthesis genes are differentially regulated in seeds compared to other tissues, we examined the expression levels of *ACS2*, *6* and *7* in different tissues at different developmental stages. While all three genes are found to be differentially expressed in different tissues, the expression levels are relatively higher in the tissues from mature plants compared to young seedling tissues. Expression levels of *ACS2*, *6* and *7* in the mature dry seeds are much lower than other tissues at mature stages (**Supplementary Figures S1A–C**). This data corresponds to our finding that ACSs are not expressed in the uninfected seeds at detectable levels, but induced to higher levels upon pathogen attack (**Figure 3**). Unlike seeds, ethylene is normally produced in the vegetative tissues, particularly at mature stages. This could explain that plants likely deploy different ACS genes to produce ET in diverse tissues and under pathogen attack.

### Colonization, Sporulation and Mycotoxin Production by *A. flavus* Grown on the *acs* Mutant Kernels

**FIGURE 4:**
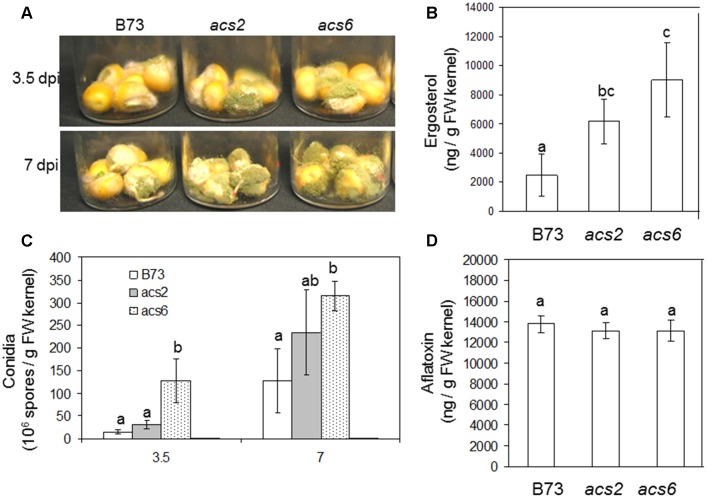
**Kernel infection bioassay of the *acs* mutants by *A. flavus*. (A)** Infected kernels of WT and *acs* mutants were cultured in glass vials and incubated for 3.5 and 7 days. **(C)** Enumeration of conidia in the *acs* mutants and WT (B73) following inoculation with *A. flavus*. **(D)** Aflatoxin and **(B)** ergosterol was measured at 7 days post-inoculation (dpi), respectively. The *acs2*, *acs6* single mutants were generated by back crossing to B73 seven times (BC_7_ stage). The values are the mean ± SD of four replicates. Different letters above bars denote significant differences (*P* < 0.05, ANOVA) analyzed by the SPSS program (SPSS Inc., Chicago, IL, USA) between the host genotypes within same time point. Similar results were obtained in three independent experiments. dpi, days post infection.

To examine the role of seed-derived ET in colonization and conidiation of *A. flavus*, we performed kernel assays using previously reported ET biosynthesis mutants that carry the *Mutator* transposons in the ACC synthase genes *ZmACS2* and *ZmACS6*, respectively (Young et al., 2004). The knockout mutants for the remaining maize ACS gene family member, *ZmACS7*, was not available for this study. The loss of *ZmACS6* expression in the *acs6* mutant resulted in a reduction of 90% of foliar ethylene, while ethylene evolution from the *acs2* mutants was only 55% of the levels produced in WT leaves (Young et al., 2004). We reasoned that these two mutants are excellent tools to investigate the role of ET in resistance to *A. flavus*. As shown in **Figure 4A**, we found that infection of both *acs2* and *acs6* mutants with *A. flavus* resulted in greater levels of seed colonization compared to WT kernels at both 3.5 and 7 dpi. Increased biomass of the fungus grown in the mutant seed was also demonstrated by increased amount of the fungus-specific lipid, ergosterol (**Figure 4B**). The enhanced colonization of the *acs* mutants was supported by increased number of conidia produced by the fungus on both mutants (**Figure 4C**). Unexpectedly, while fungal vegetative growth on *acs2* and *acs6* mutants was increased, aflatoxin B1 levels remained similar in all maize genotypes (**Figure 4D**). These results suggest that the *ACS2* and *ACS6* genes function in maize responses to *A. flavus* colonization and spore production but are irrelevant to the fungal ability to produce aflatoxin.

### ET Production Is Induced in the *acs* Mutants Infected with *A. flavus*

Expression pattern of ET biosynthesis genes after inoculation with *A. flavus* (**Figure 3**) and greater colonization of *acs* mutant kernels by *A. flavus* (**Figure 4**) suggested that ET could be a key factor in the regulation of seed colonization by *A. flavus*.

**FIGURE 5:**
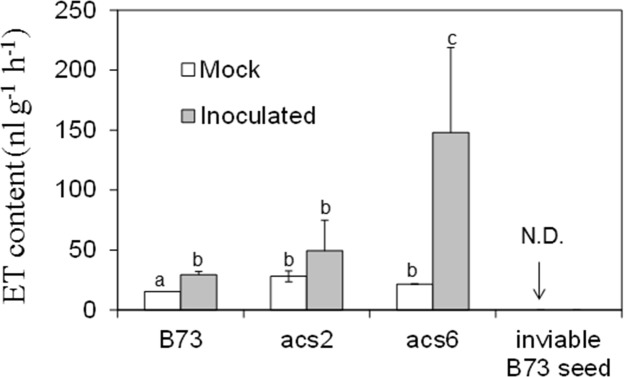
**Ethylene production on *acs* mutants upon inoculation with *A. flavus NRRL* 3357.** The kernels were inoculated with 1 × 10^6^ spores and cultured in a 20 ml of glass scintillation vial (Wheaton Science) at 29°C under 12L/12D for 7 days. The vial was then tightly sealed with a plastic lid for 1 to 2 h prior to analysis to allow enough ethylene was accumulated. ET was measured by withdrawing 1 mL volume of headspace gas with a syringe and injecting into a digital gas chromatograph (Photovac 10 plus, PerkinElmer, Inc., Norwalk, CT, USA) The values are the mean ± SD of four replicates (four vials each treatment each genotype), with six kernels in each vial. Different letters above bars denote significant differences (*P* < 0.05, ANOVA) analyzed by SPSS program (SPSS Inc., Chicago, IL, USA) between the host genotypes within same time point. Similar results were obtained in three independent experiments. dpi, days post infection. N.D., not detectable.

To confirm that *acs* mutants are true knock-out alleles, we measured ET levels in B73 and *acs6* mutant leaves. As shown in **Supplementary Figure S1D**, almost no ET was detected in the leaves of the *acs6* mutant, confirming the mutant is indeed a true knock-out mutant. To further test whether ET production is altered in kernels of the *acs* mutants in response to *A. flavus* infection, we measured ET emission in WT and the *acs* mutants kernels in response to infection by *A. flavus*. ET was quantified from the headspace of the vials containing WT or the *acs* mutant kernels at 7 dpi. While infected WT kernels emitted only slightly greater levels of ET, unexpectedly, ET levels were strongly enhanced in the kernels of both *acs2* and *acs6* mutants, with the significantly higher levels in *acs6* mutants (**Figure 5**). This is in sharp contrast to the reported reduced ET production levels in the leaves of the two mutants (Young et al., 2004). Non-infected desiccated seed of the mutants or WT did not emit any detectable ET (data not shown).

### Seed but not the Fungus Is Responsible for Induced ET Emission

Because the *acs2* and *acs6* infected seed emitted greater than WT levels of ET, we tested whether ET was produced by *A. flavus* rather than by the host (or in addition to the host). We reasoned that autoclaved, and thus non-viable, seed will not be able to synthesize ET, while the fungus that colonizes such seed may produce ET. In contrast to viable seed, no ET emission could be detected when non-viable kernels were infected by *A. flavus* (**Figure 5**). These data suggest that ET is originated from maize host but not the fungus.

### Effects of an ET Inhibitor on the Colonization and Sporulation of *A. flavus*

**FIGURE 6:**
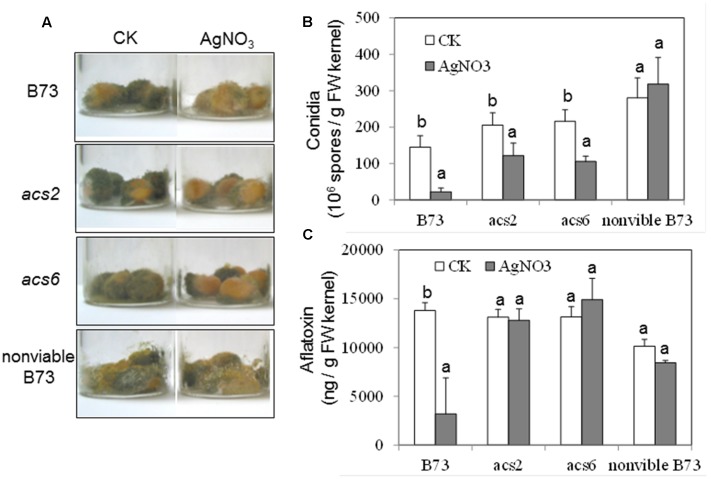
**Effect of ethylene inhibitor AgNO_3_ on the colonization, conidiation and aflatoxin production of *A. flavus NRRL* 3357 on *acs* mutants. (A)** The kernels were pretreated with AgNO_3_ (20 mM) 30 min, followed by inoculation with 1 × 10^6^ spores, then cultured in a 20 ml of glass scintillation vial (Wheaton Science) at 29°C under 12L/12D for 7 days. **(B)** Conidiation of *acs* mutants upon infection of *A. flavus NRRL* 3357. The values are the mean ± SD of four replicates (four vials each treatment each genotype), with six kernels in each vial. **(C)** Aflatoxin B1 was quantified using Vicam Aflatest (Vicam, Watertown, MA, USA), according to the USDA-FGIS protocol. The values are the mean ± SD of four replicates (four vials each treatment each genotype), with six kernels in each vial. Different letters above bars denote significant differences (*P* < 0.05, ANOVA) analyzed by SPSS program within same host genotypes. Similar results were obtained in at least three independent experiments. dpi, days post infection.

To confirm the role of ET in the kernel-*A. flavus* interaction by a pharmacological approach, the potent ET inhibitor, AgNO_3_, was applied to the kernels prior to inoculation. The pretreatment with the inhibitor reduced fungal colonization of both WT and the single *acs* mutants, as evidenced by the reduced number of conidia (**Figures 6A,B**). In the WT kernels, the inhibitor pretreatment resulted in reduced production of aflatoxin, which correlated with the lower fungal growth (**Figure 6C**). However, pretreatment with the inhibitor did not appear to have an effect on the levels of aflatoxin in both *acs* mutants (**Figure 6C**).

We also tested whether the inhibitor treatment had any unintended effect on the ability of *A. flavus* to colonize seed, and produce conidia and aflatoxin in the infected, non-viable seeds. As shown in **Figure 6A**, the ability of *A. flavus* to colonize kernels and to produce conidia was not affected by AgNO_3_ in the non-viable seeds, compared to control non-treated seeds (**Figures 6A,B**). These data suggested that AgNO_3_ treatment itself unlikely affected fungal development and secondary metabolism (e.g., toxin production) directly, and that the inhibitor effects observed on the living seed were due to the suppression of ET effect in the host seed.

To test whether ET precursor, ACC, could itself promote pathogenicity of *A. flavus* in maize kernels, we pretreated kernels of B73, *acs2* and *acs6* mutant seeds with ACC, followed by inoculation with *A. flavus*. While we found that both *acs2* and *acs6* displayed enhanced conidiation of *A. flavus* on mutant kernels compared to B73, ACC treatment showed increased but not statistically significantly higher number of conidia produced by *A. flavus*, compared to the mock treatment (**Supplementary Figures S2A,B**). However, pretreatment with ACC treatment could significantly increase the ergosterol content in B73 seeds, compated to the control (**Supplementary Figure S2C**), supporting our findings that ET might serve as a susceptibility factor for fungal growth. The effect of ACC on ergosterol was not observed in either *acs* mutant. This could be due to possible saturation of the seed capability of converting ACC to yet additional ET in the acs mutant kernels in response to *A. flavus*.

### Expression of ET Biosynthetic Genes in WT and *acs6* Mutants upon Infection with *A. flavus*

**FIGURE 7:**
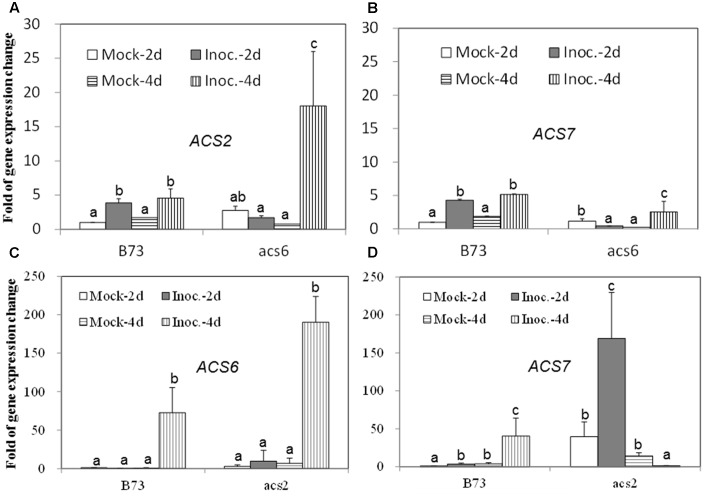
**The expression of ET biosynthetic genes, *ACS2, ACS6* and *ACS7*, in the WT and *acs* mutant kernels infected with *A. flavus*.** The kernels were inoculated with 1 × 10^6^ spores and cultured in a 20 ml of glass scintillation vial (Wheaton Science) at 29°C under 12L/12D for 2 and 4 days, and gene expression levels of *ACS2* **(A)** and *ACS7* **(B)** in B73 vs. *acs6* mutants, and *ACS6* **(C)** and *ACS7* **(D)** in B73 vs. *acs2* mutants, respectively, were quantified using qRT-PCR. Different letters above bars denote significant differences (*P* < 0.05, ANOVA) analyzed by the SPSS program between different treatments within the host genotypes. Similar results were obtained in three independent experiments.

**FIGURE 8:**
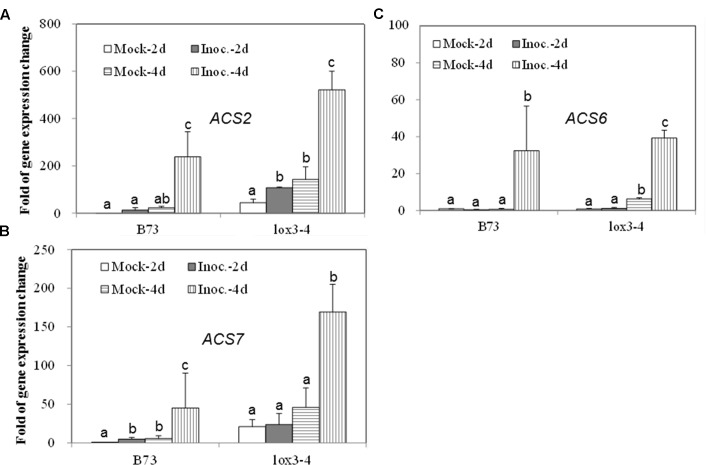
**The expression of ET biosynthetic genes, *ACS2, ACS6* and *ACS7*, in the WT and *lox3-4* mutant kernels infected with *A. flavus*.** The kernels were inoculated with 1 × 10^6^ spores and cultured in a 20 ml of glass scintillation vial (Wheaton Science) at 29°C under 12L/12D for 2 and 4 days, and gene expression levels of *ACS2* **(A)**, *ACS6* **(B)** and *ACS7* **(C)** in B73 vs. *lox3-4* mutants, respectively, were quantified using qRT-PCR. Different letters above bars denote significant differences (*P* < 0.05, ANOVA) analyzed by the SPSS program between different treatments within the host genotypes. Similar results were obtained in three independent experiments.

Since the *acs* mutants, produced more ET compared to WT, in response to the infection with *A. flavus*, we hypothesized that the increased ET levels observed in the single mutants might be due to the overexpression of other *ACS* genes. As shown in **Figures 7A,B**, the expression of both *ACS2* and *ACS6* was moderately increased in WT seed upon *A. flavus* infection, while *ACS2* expression, but not *ACS7*, was strongly induced to a much higher levels in the *acs6* mutant compared to that in WT seeds, at 4 dpi. In the *acs2* mutant seed, *ACS6* expression was induced to a higher level upon infection, compared to that in WT seed (**Figure 7C**), at 4 dpi. However, the expression of *ACS7* was enhanced earlier, at 2 dpi, in the *acs2* mutant seed, compared to that in WT, where it was induced to a higher level at 4 dpi (**Figure 7D**). In support of this finding, the expression of both *ACS2* and *ACS7* was significantly higher in the *lox3-4* mutants after infection with *A. flavus* at 4 dpi, compared to that in WT (**Figures 8A,B**). Although the expression of *ACS6* was also strongly increased in *lox3-4* mutant, however, that numerical increase was not statistically different (**Figure 8C**).

## Discussion

*Aspergillus flavus* colonizes the same niche, i.e., maize kernels, but is evolutionarily and ecologically distinct from *Fusarium* spp. Kernel assays showed consistently that *acs* mutants were more susceptible to *A. flavus*. The increased colonization and sporulation of *A. flavus* on *acs* mutant kernels was associated with the enhanced ET production, especially in *acs6* (**Figure 5**). Here we also demonstrated that susceptible to *Aspergilli* spp. *lox3-4* mutant (Gao et al., 2009), also produced greater levels of ET upon infection with *A. flavus* compared to WT and provides a support for presumptive role of ET in facilitating *A. flavus* pathogenesis (**Figure 1**). Coincidently, *lox3-4* mutant was also reported to produce greater levels of ET in the roots, suggesting that 9-oxylipins produced by the LOX3 isoform are potent inhibitors of ET synthesis in both seeds and roots.

Taken together, the findings from this study suggest that seed derived ET facilitate seed colonization and conidiation by *A. flavus*. Our results did not provide conclusive evidence as to whether host-derived ET has a role in the regulation of aflatoxin biosynthesis. While fungal colonization and spore production by *A. flavus* were increased in the *acs* mutant kernels, aflatoxin production was not impacted in *acs2* or *acs6* (**Figure 4**), suggesting that ET function differentially in the regulation of fungal growth and secondary metabolite production. It appears, however, that the increased colonization of *acs* mutants may be responsible for the same level of aflatoxin produced by relatively lower fungal biomass in WT seed.

It was intriguing to find that seed-derived ET upon the infection stimulates the growth of *A. flavus*, whereas did not show any impact on aflatoxin production in corn seeds. This contrasts with the studies showing that exogenously applied ET could inhibit aflatoxin production by *A. parasiticus* in peanuts (Roze et al., 2004; Gunterus et al., 2007; Huang et al., 2009). ET and its generator, ethephon, also suppressed aflatoxin biosynthesis in *A. flavus in vitro*, which might be due to ROS reduction (Huang et al., 2009). There are three possible reasons for this discrepancy. First, the amount of seed-derived endogenous ET that we detected is within the range of nl/L, which was far below the amount of ET that previous studies used (1∼100ppm, within a range of ul/L) (Roze et al., 2004; Gunterus et al., 2007; Huang et al., 2009). Another possible reason is that endogenous ET at low threshold produced by host upon infection could serve as signaling molecule facilitating *A. flavus* vegetative growth, but not aflatoxin synthesis. There is also a possibility that ET role in the pathogenicity is host-dependent (peanut vs. maize). Further studies are required to investigate the precise function and mechanisms of ET in different pathosystems.

One of the intriguing findings of this study is that ET production in response to *A. flavus* infection was not reduced, as expected, in both *acs2* and *acs6* mutant kernels (**Figure 5**). On the contrary, ET was increased in *acs2* and even higher in *acs6* mutants. Further analyses of why the single mutants produce greater levels of ET revealed that they overexpress other members of the ACS gene family. Maize *ACS* gene family consists of at least three members, *ZmACS2*, its closely related paralog *ZmACS7*, and a distantly related *ZmACS6* (Gallie and Young, 2004; Young et al., 2004). *ACS2* and to a lesser extent *ACS7* transcript abundance was enhanced in the *acs6* mutant seed upon the infection with the fungus, and *acs2* mutant overexpressed the *ACS6* gene. Interestingly, it has been shown in an unrelated study that maize *acs6* mutants overexpress *ZmACS2* gene while expression of the *ZmACS7* gene was reduced (Young et al., 2004). Similar to our finding that single *acs* mutants overproduce ET, previous study showing that the Arabidopsis *eto2* mutant, which produces 20-fold greater levels of ET, is deficient in the *ACS5* gene expression supports the idea of antagonistic interaction between ET-producing enzyme isoforms or the ability of the functional genes to compensate for the lack of a missing gene family member (Vogel et al., 1998).

Previous reports showed that *A. parasiticus* produces ET on solid culture medium (Roze et al., 2004). In this study, however, no fungus-derived ET was detected in either fungal culture plates (data not shown) or from the fungus grown on non-viable seeds (**Figure 5**), suggesting that *A. flavus* is not the source of increased ET in the assays with living seeds. This discrepancy may be explained by different timing of ET sampling. For example, it has been reported that *A. flavus* produced much less ET compared to *A. parasiticus*, and the emission of ET by the former occurred at the early period of their growth, after which the ET level declined to the level that was not detectable in the system (Sharma et al., 1985). Alternatively, even if *A. flavus* produces ET when grown on a medium, this production may be inhibited on a plant tissue. This scenario was reported in *B. cinerea*-tobacco pathosystem, where *B. cinerea* produced easily detectable levels of ET *in vitro* but did not produce any ET *in planta* (Chague et al., 2006).

Our findings in this study provide strong evidence that ET biosynthetic enzymes *ZmACS2* and *ZmACS6* and their final product ET directly or indirectly play a major role in governing the outcomes of seed interactions with the mycotoxin-producing fungi. The phenotypes of the two mutants resemble closely the disease phenotypes previously reported for the 9-lipoxygenase mutant, *lox3-4*, except that *F. verticillioides* grew equally well on both the *lox3-4* mutant and B73, but produced up to 200-fold lower fumonisin and threefold lower conidia levels (Gao et al., 2007). Supporting the hypothesis that ET may facilitate fungal growth and conidia production is our finding that *A. flavus-*infected *lox3-4* mutants emitted greater levels of ET compared to WT (**Figure 1**). Similar to ET positive correlation with fungal growth and conidiation shown in this study, in the *lox3-4* studies increased colonization by *A. flavus* of *lox3-4 mutant* and greater spore and mycotoxin production by *F. verticillioides* grown on WT kernels correlated with the increased accumulation of fatty acids (Gao et al., 2009). Therefore, we propose that both fungi exploit not only ET but also lipids to facilitate their virulence. It is possible that the two groups of signals, *ZmLOX3*-dependent lipid-derived molecules and *ZmACS2/6*-dependent ET, may act synergistically or are interdependent. The precise mechanism of their interactions, if any, is not clear and needs to be further examined by using the *lox3* and the *acs* double and triple mutants being constructed in our laboratories.

## Distribution Of Materials

Novel materials described in this publication may be available for non-commercial research purposes upon acceptance and signing of a material transfer agreement. In some cases such materials may contain or be derived from materials obtained from a third party. In such cases, distribution of material will be subject to the requisite permission from any third-party owners, licensors, or controllers of all or parts of the material. Obtaining any permission will be the sole responsibility of the requestor. Plant germplasm will not be made available except at the discretion of the owner and then only in accordance with all applicable governmental regulations.

## Author Contributions

XG and MK designed the research. SW, Y-SP, YY, TI, and XG performed research. SW, Y-SP, EB, XG, and MK performed data analysis. Y-SP, XG, and MK drafted the article. XG and MK performed critical revision of the article. XG and MK carried out final approval of the final version.

## Conflict of Interest Statement

The authors declare that the research was conducted in the absence of any commercial or financial relationships that could be construed as a potential conflict of interest.

## Supplementary Material

FIGURE S1 **Expression levels of *ACS2*, *ACS6* and *ACS7* in different maize organs from different growth stages (A–C)**, and ET levels in the leaves of *acs6* mutant **(D)**. Expression levels of *ACS2* **(A)**, *ACS6* **(B)** and *ACS7* **(C)** was examined by qRT-PCR in different maize organs from different growth stages. Eg, embryo from geminating seeds (2-day-old); Rg, root from geminating seeds (4-day-old); R1, root at V1 stage; S1, stem at V1 stage; L1, leaf at V1 stage; R3, root at V3 stage; S3, stem at V3 stage; L3, leaf at V3 stage; Rf, root at flowering time; Sf, stem at flowering time; Lf, leaf at flowering time; T, tassel; E, ear; P, pollen; K, kernel. **(D)** ET levels in the leaves of B73 and *acs6* mutant. The plants were grown as described in Section “Materials and Methods.” The leaves of B73 and acs6 were excised from the plants and immediately transferred into a 20 ml of glass scintillation vial (Wheaton Science). The vial was then tightly sealed with a plastic lid for 1 to 2 h prior to analysis to allow sufficient levels of ethylene to accumulate. ET was measured by gas chromatograph as described. The values are the mean ± SD of four replicates (four vials each treatment each genotype), with three leaf segments in each vial.

FIGURE S2 **Effect of ethylene precursor ACC on the colonization, conidiation, and ergosterol production by *A. flavus* NRRL 3357 on *acs* mutants. (A)** The kernels were pretreated with ACC (20 nM) for 30 min, followed by inoculation with 1 × 10^6^ spores, then cultured in a 20 ml of glass scintillation vial (Wheaton Science) at 29°C under 12L/12D for 7 days. CK is the control by pretreatment with same amount of 0.001% Tween-20 as that used for inoculum of *A. flavus*. **(B)** Conidiation observed on the *acs* mutants upon infection of *A. flavus NRRL* 3357. The values are the mean ± SD of at least four replicates (four vials each treatment each genotype), with four kernels in each vial. Different letters above bars denote significant differences (*P* < 0.05, ANOVA) analyzed by SPSS program over B73-CK. Similar results were obtained in two independent experiments. **(C)** Ergosterol content was measured at 7 days post-inoculation (dpi).
