# Supplementary material for: Seed-Derived Ethylene Facilitates Colonization but Not Aflatoxin Production by Aspergillus flavus in Maize
Source: Front Plant Sci. 2017 Mar 28;8:415. doi: 10.3389/fpls.2017.00415 (PMC5368243; doi:10.3389/fpls.2017.00415)
Supplement: Supplementary file 1 [file Table_1.docx]

**Supplemental Table 1. Primers used in this study**

| Primer ID | Sequences |
| --- | --- |
| ZmACS2-qRT-F1 | 5’-CGCCATACTACCCAGCTTTC-3’ |
| ZmACS2-qRT-R1 | 5’CGTGTGAGGGTGAAGTTGTT-3’ |
| ZmACS7-qRT-F1 | 5’ACGCCTTATTACCCAGCTTTC-3’ |
| ZmACS7-qRT-R1 | 5’CTGGTGAGGGTGAAGTTGTT-3’ |
| ZmACS6-qRT-F4 | 5’TCATCACCAACCCTTCCAAC-3’ |
| ZmACS6-qRT-R4 | 5’AGTATATCTCGTCGCTCACCA-3’ |
| beta -TUB-F | 5’CTACCTCACGGCATCTGCTATGT-3’ |
| beta-TUB-R | 5’GTCACACACACTCGACTTCACG-3’ |
