# Supplementary figures and images for: Seed-Derived Ethylene Facilitates Colonization but Not Aflatoxin Production by Aspergillus flavus in Maize
Source: Front Plant Sci. 2017 Mar 28;8:415. doi: 10.3389/fpls.2017.00415 (PMC5368243; doi:10.3389/fpls.2017.00415)

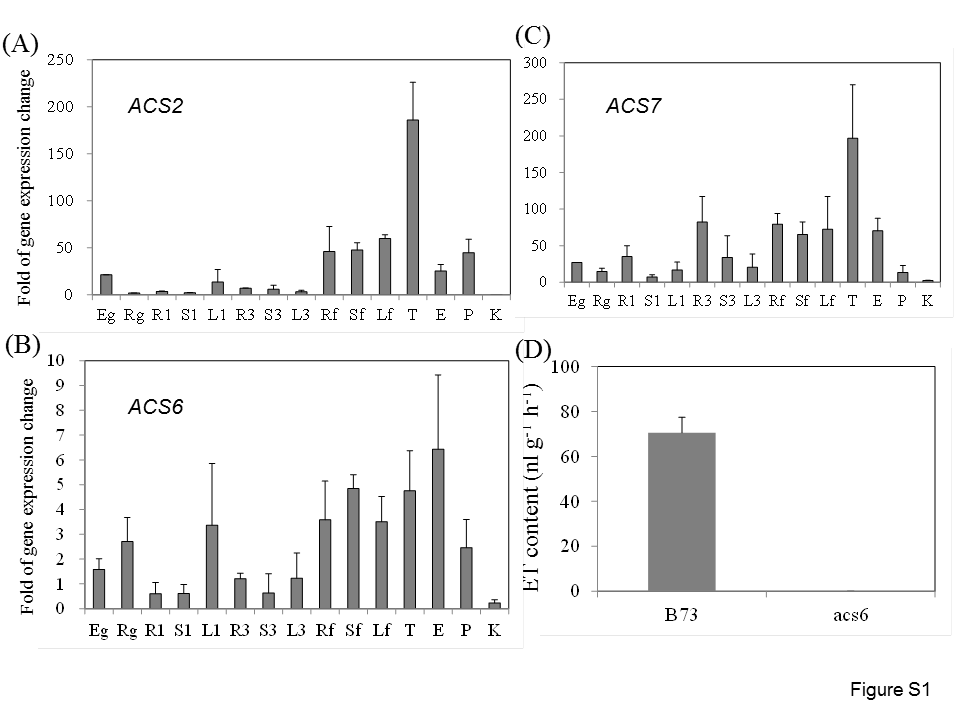

Supplement: FIGURE S1 — Expression levels of ACS2, ACS6 and ACS7 in different maize organs from different growth stages (A–C), and ET levels in the leaves of acs6 mutant (D). Expression levels of ACS2 (A), ACS6 (B) and ACS7 (C) was examined by qRT-PCR in different maize organs from different growth stages. Eg, embryo from geminating seeds (2-day-old); Rg, root from geminating seeds (4-day-old); R1, root at V1 stage; S1, stem at V1 stage; L1, leaf at V1 stage; R3, root at V3 stage; S3, stem at V3 stage; L3, leaf at V3 stage; Rf, root at flowering time; Sf, stem at flowering time; Lf, leaf at flowering time; T, tassel; E, ear; P, pollen; K, kernel. (D) ET levels in the leaves of B73 and acs6 mutant. The plants were grown as described in Section “Materials and Methods.” The leaves of B73 and acs6 were excised from the plants and immediately transferred into a 20 ml of glass scintillation vial (Wheaton Science). The vial was then tightly sealed with a plastic lid for 1 to 2 h prior to analysis to allow sufficient levels of ethylene to accumulate. ET was measured by gas chromatograph as described. The values are the mean ± SD of four replicates (four vials each treatment each genotype), with three leaf segments in each vial. [file Image_1.TIF]

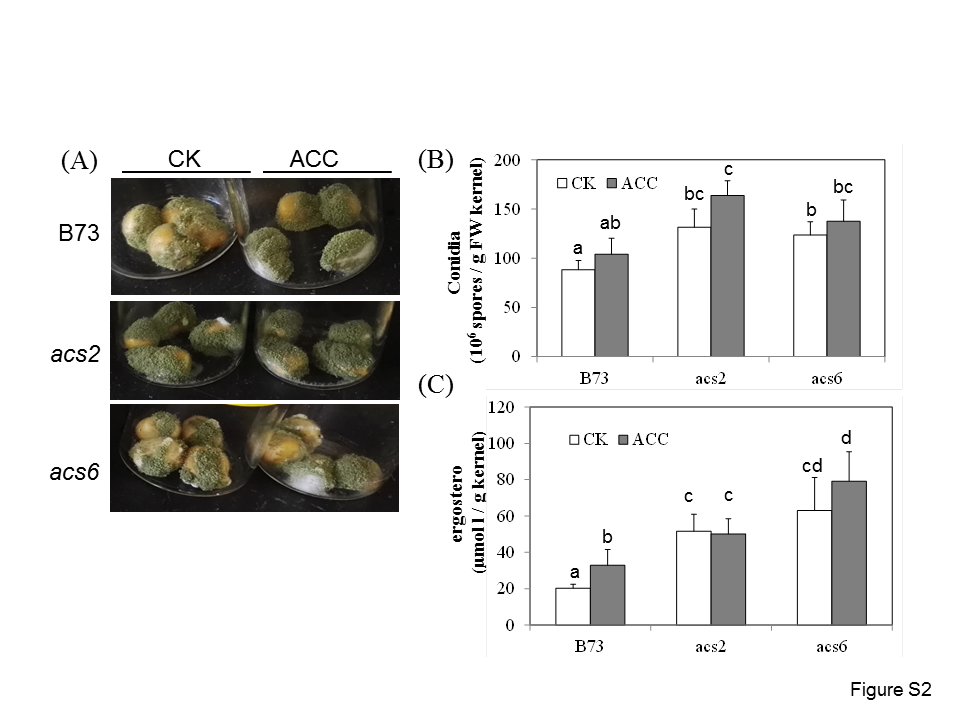

Supplement: FIGURE S2 — Effect of ethylene precursor ACC on the colonization, conidiation, and ergosterol production by A. flavus NRRL 3357 on acs mutants. (A) The kernels were pretreated with ACC (20 nM) for 30 min, followed by inoculation with 1 × 10^6^ spores, then cultured in a 20 ml of glass scintillation vial (Wheaton Science) at 29°C under 12L/12D for 7 days. CK is the control by pretreatment with same amount of 0.001% Tween-20 as that used for inoculum of A. flavus. (B) Conidiation observed on the acs mutants upon infection of A. flavus NRRL 3357. The values are the mean ± SD of at least four replicates (four vials each treatment each genotype), with four kernels in each vial. Different letters above bars denote significant differences (P < 0.05, ANOVA) analyzed by SPSS program over B73-CK. Similar results were obtained in two independent experiments. (C) Ergosterol content was measured at 7 days post-inoculation (dpi). [file Image_2.TIF]
